# Applications and Performance of Precision ID GlobalFiler NGS STR, Identity, and Ancestry Panels in Forensic Genetics

**DOI:** 10.3390/genes15091133

**Published:** 2024-08-28

**Authors:** Sharlize Pedroza Matute, Sasitaran Iyavoo

**Affiliations:** 1 School of Natural Sciences, University of Lincoln, Brayford Pool, Lincoln LN6 7TS, UK; 2 AttoGroup Limited, Scottow Enterprise Park, Badersfield, Norwich NR10 5FB, UK

**Keywords:** Precision ID, HID Ion AmpliSeq, short tandem repeats, GlobalFiler, single-nucleotide polymorphisms, ancestry SNPs, identity SNPs, next-generation sequencing, massively parallel sequencing, forensic genetics

## Abstract

Short Tandem Repeat (STR) testing via capillary electrophoresis is undoubtedly the most popular forensic genetic testing method. However, its low multiplexing capabilities and limited performance with challenging samples are among the factors pushing scientists towards new technologies. Next-generation sequencing (NGS) methods overcome some of these limitations while also enabling the testing of Single-Nucleotide Polymorphisms (SNPs). Nonetheless, these methods are still under optimization, and their adoption into practice is limited. Among the available kits, Thermo Fisher Scientific (Waltham, MA, USA) produces three Precision ID Panels: GlobalFiler NGS STR, Identity, and Ancestry. A clear review of these kits, providing information useful for the promotion of their use, is, however, lacking. To close the gap, a literature review was performed to investigate the popularity, applications, and performance of these kits. Following the PRISMA guidelines, 89 publications produced since 2015 were identified. China was the most active country in the field, and the Identity Panel was the most researched. All kits appeared robust and useful for low-quality and low-quantity samples, while performance with mixtures varied. The need for more population data was highlighted, as well as further research surrounding variables affecting the quality of the sequencing results.

## 1. Introduction

Since the discovery of DNA fingerprinting in the 1980s, the field of forensic genetics has evolved with new markers and technologies. To date, the most popular forensic identification method relies on the analysis of Short Tandem Repeat (STR) markers via capillary electrophoresis (CE), which was introduced into routine use in the mid-1990s [1,2]. STRs are powerful markers for forensic identification, with applications in paternity and maternity testing, more complex kinship testing, and ancestry analysis [3,4,5]. However, due to inherent characteristics of STRs, such as their length, abundance, and mutation rates, and the limitations of CE, including restricted multiplexing capabilities, new markers and technologies have begun to be investigated [6,7,8].

In 2011, the possibility of applying next-generation sequencing (NGS), also referred to as massively parallel sequencing (MPS), to the forensic field started to emerge [1]. By generating the sequence of the targeted markers, the application of NGS technologies to STR typing offers multiple advantages. Compared to CE, an increased number of markers can be processed simultaneously, and the length of the targeted fragments can be reduced. This reduction improves the method’s efficacy when dealing with degraded and low-quality DNA, which is often encountered at crime scenes. Additionally, the availability of sequence information can help distinguish stutter artefacts and can unveil increased diversity both within the repeat sequence and in the locus flanking region, leading to higher discrimination power [1,6]. Moreover, NGS technologies can genotype Single-Nucleotide Polymorphisms (SNPs). SNPs are markers with a short size, ideal for degraded samples and multiplexing, and with a lower mutation rate than STRs, which is advantageous for kinship and ancestry testing. Some SNPs have also been found to be associated with physical characteristics, including eye, hair, and skin color, allowing for the possibility of gathering phenotypical information from unknown DNA. Even though SNPs are less polymorphic than STRs, their high abundance throughout the genome makes them useful markers for identification purposes as well [7]. According to their use, SNPs have been classified into four categories: identity, lineage, ancestry informative, and phenotype informative [9]. A novel type of markers consisting of two or more closely linked, associated SNPs present in a region of less than 300 nucleotides has also recently emerged. These are called ‘microhaplotypes’ and are arising as promising markers in multiple forensic genetics applications, including human identification, mixture deconvolution, and biogeographic ancestry inference [10].

Different types of NGS technologies have been developed so far. While validations for forensic use are ongoing for other manufacturers, Illumina and Thermo Fisher Scientific are leading the market with the MiSeq/NextSeq platforms and Ion Torrent Personal Genome Machine (PGM)/Ion S5 instruments, respectively. A 2017 survey showed that more than half of 33 European forensic laboratories already owned NGS instruments, with MiSeq being the most popular (10 instruments), followed by Ion PGM (7 instruments) and Ion S5 (4 instruments) [11]. A more recent 2020 survey from 32 European countries confirmed the growing popularity of NGS in forensics, with 73% of 105 institutions already owning (46%) or planning to acquire (27%) MPS instruments. Out of the 64 instruments reported in the survey, the same percentage of MiSeq/MiSeq FGx and Ion PGM/Ion S5 was observed, with only 6.3% for other instruments [12].

The MiSeq FGx platform, manufactured by the Illumina-established company Verogen and later acquired by Qiagen, was the first sequencing platform developed and validated for forensic genomics. The instrument was specifically designed to be used with the ForenSeq DNA Signature Prep Kit [13,14]. This kit includes the sex-determining marker Amelogenin, 27 common autosomal STRs, 24 Y-chromosome STRs, 7 X-chromosome STRs, and an option for three classes of SNPs, including 94 identity-informative SNPs, 22 phenotypic-informative SNPs, and 56 ancestry SNPs, for a total of 231 markers [15]. Multiple validations, assessments with degraded samples, use in distant kinship analysis, and multiple other studies were performed on this kit, indicating widespread use [16,17,18,19,20]. The ForenSeq kit was employed to process thousands of samples from different nationalities to populate the STRSeq GenBank NCBI BioProject, a sequencing project aimed at providing population data for STR sequencing [21].

The Ion Torrent PGM and S5 are the most used Thermo Fisher Scientific’s platforms for forensic investigations. Among the kits developed for these instruments, the Precision ID GlobalFiler NGS STR Panel version 2 contains 35 markers, 23 of which are included in the GlobalFiler kit developed for CE [13,22]. The CE GlobalFiler kit contains the 13 original Combined DNA Index System (CODIS) core loci and 7 non-overlapping loci from the expanded European Standard Set (ESS) (20 expanded CODIS core loci), the highly discriminating SE33 locus, two Y-chromosome markers, and Amelogenin [23]. The Precision ID GlobalFiler NGS STR Panel excludes SE33, one of the most polymorphic forensic markers for human identification, likely due to the bioinformatic challenges of analyzing its sequence [24]. The NGS STR Panel also includes additional 12 forensically relevant markers, for a total of 31 autosomal markers, Amelogenin, and three Y markers [22]. It is supplied with integrated data analysis using Converge Software, which enables not only analysis of STR results, but also comparisons with CE results, SNP analysis, ancestry estimation, and calculation of statistical support for paternity and kinship cases [25].

Apart from the GlobalFiler kit, the Precision ID NGS System includes additional panels with potential forensic application: the Precision ID Identity Panel, the Precision ID Ancestry Panel, and the two mitochondrial kits; mtDNA Whole Genome and mtDNA Control Region [26]. The Precision ID Identity Panel, formerly known as the HID Ion AmpliSeq Identity Panel, includes 124 SNPs, 34 upper Y-clade SNPs, and 90 autosomal SNPs with high discrimination power, with short amplicons that render them ideal for challenging samples. The autosomal SNPs are the result of the combination of previous publications; 43 SNPs from the Kidd panel and 48 from the SNPforID Consortium SNPlex system (one overlapping SNP) [26,27,28]. The Y SNPs are useful markers for defining Y haplogroups, paternally inherited polymorphisms that tend to be shared between related male individuals and within specific geographical areas. These characteristics make them valuable not only for identification and relationship testing of male DNA, but also for biogeographic inference studies [29]. The Precision ID Ancestry Panel, formerly known as HID Ion AmpliSeq Ancestry Panel, was developed specifically for biogeographic inference purposes. It contains 165 autosomal SNPs selected to provide biogeographic ancestry information, with small amplicon size (<130 bp) to allow processing of degraded samples. These SNPs were selected from two panels, 55 from the Kidd ancestry panel and 123 from the Seldin panel (13 overlapping SNPs) [26,30,31].

Even though certain panels may share the same markers, some discordance was observed between the results obtained across different platforms [32]. Diverse performance was also noted when using different instruments, including distinct resistance to inhibitors [33]. To enhance understanding and increase confidence in NGS results, it is important to continue testing and exploring the pros and cons of different kits and platforms. In fact, despite the benefit of NGS techniques, their application in routine forensic practice is still lacking. Data analysis can be challenging and requires specialized knowledge, while databases necessitate continuous efforts to expand. On top of this, setup and running costs are prohibitive for many laboratories, while nomenclature and reporting standards are still being defined. Further research and the development of guidelines are therefore needed to support the introduction of NGS methods into routine use and improve confidence in the generated results [1,6,11].

The aim of this review was to better understand the current application of the Thermo Fisher Scientific Precision ID GlobalFiler NGS STR, Identity, and Ancestry panels for forensic purposes, to investigate how popular they are, where and how they are more prevalently researched, and to inspect their strengths and weaknesses. By evaluating their application and performance, we aim to highlight areas that require further study to help promote the progressive introduction of NGS technologies into routine forensic use.

## 2. Materials and Methods

The articles presented in this review were selected following the PRISMA guidelines [34]. The search engines used were Web of Science (WoS), Scopus, and PubMed. The following search terms were used: ‘Precision ID GlobalFiler NGS STR Panel’, ‘Precision ID GlobalFilerTM NGS STR Panel’, ‘Precision ID Identity Panel’, ‘HID Ion AmpliSeq Identity Panel’, ‘HID Ion AmpliSeqTM Identity Panel’, ‘Precision ID Ancestry Panel’, ‘HID Ion AmpliSeq Ancestry Panel’, and ‘HID Ion AmpliSeqTM Ancestry Panel’. The search terms were queried by ‘Topic’ from ‘All Databases’ for WoS, which searches title, abstract and indexing, by ‘Article title, Abstract, Keywords’ for Scopus, and by ‘All Fields’ for PubMed. To obtain all the relevant literature produced until the review date (May 2024), no date restrictions were applied.

**Figure 1:**
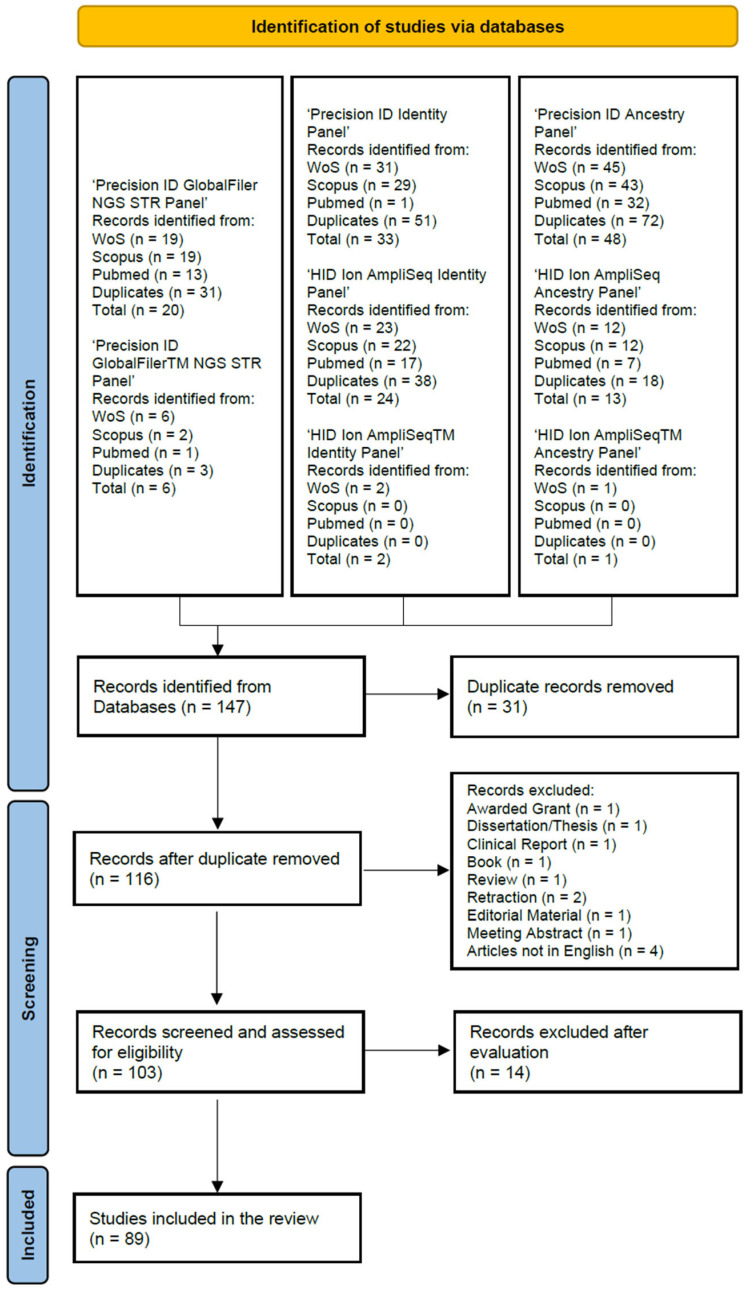
Flow diagram delineating the records selection process for this review.

Duplicates were removed from the articles found. Records that were not peer-reviewed scientific publications, including awarded grants, dissertations, clinical reports, books, editorial material, and meeting abstracts, were excluded. Reviews, retracted articles, and articles in languages other than English were also excluded as part of the initial screening. Subsequently, the abstracts and content of the articles were examined to exclude any publication that was not relevant to this study’s aim. Only articles focused on the use of the Precision ID/HID Ion AmpliSeq kits under consideration or that used the same SNP panels were retained. Articles covering the use of the Precision ID mitochondrial kits, articles that did not refer to the kits under investigation, or articles that merely mentioned the kit without further evaluation or comparison were excluded. A flow diagram of this studies identification process based on the PRISMA guidelines template is provided in Figure 1.

The 89 articles obtained after applying these inclusion and exclusion criteria were thoroughly examined, considering factors such as publication year, first author affiliations, journal, article type, access restrictions, and article topic. Relevant graphs were generated using Excel version 2407.

## 3. Results

**Figure 2:**
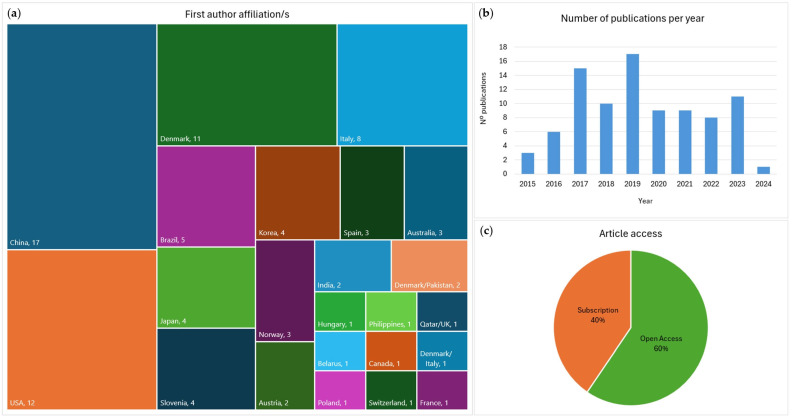
(**a**) TreeMap indicating the number of studies published from each originating affiliated country; (**b**) bar chart indicating the number of articles published each year; (**c**) pie chart showing the percentage of articles published with open rather than restricted access.

As seen in Figure 2a, most of the publications covering the use of the kits under consideration were generated by research groups located in China (19%), followed by the USA (13%), Denmark (12%), and Italy (9%). When accounting for authors with multiple affiliations, 15% of the articles were linked to Danish and 10% to Italian research groups. When investigating the contribution by continent, 42% of the articles originated from Europe, 31% from Asia, and 15% from North America. Of the 89 records identified, the first articles were published in 2015, and since then, an average of nearly nine articles were published each year. The most publications were produced in 2019 (19%), and the fewest in 2024 (1%), for which only publications produced until May were considered (Figure 2b). Forty percent of all records were available as open access, while the remaining were available only through subscription (Figure 2c).

**Figure 3:**
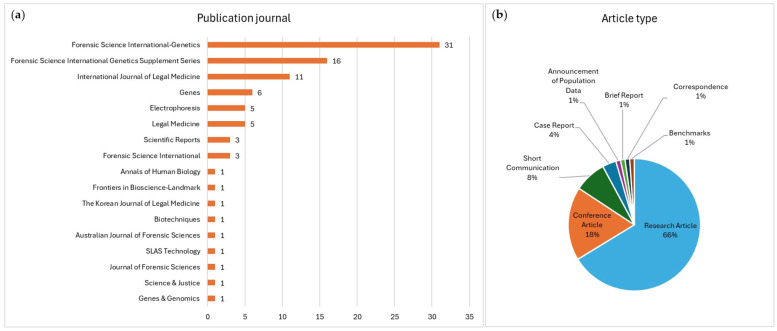
(**a**) Number of articles published per journal; (**b**) percentage of articles belonging to each article type observed.

Overall, more than half the articles were published in *Forensic Science International*: the *Genetics* (35%) and *Supplement Series* (18%), with 80% of the total records published in the same eight journals (Figure 3a). The *Supplement Series* journal collates publications from proceedings of scientific symposiums and a selection of invited articles, which are categorized here as ‘Conference Article’. Sixty-six percent of the records were categorized as ‘Research Article’, with only a small percentage classified as ‘Short Communication’, ‘Case Report’, and other categories, as indicated in Figure 3b.

The sequencing panel and instrument used, along with a summary of the main points for each article included in this review, are summarized in Table 1.

Table 1 highlights the presence of articles produced by the same main authors. For example, both Pajnič and Wang were, separately, among the first authors in four articles, while 18 individuals appeared as main authors in two publications. Also, several articles emerged as probable expansions on previous work first published, for example, as a conference article, and reproposed with additions as full research articles. These include, for instance, Pajnič, Pogorelc, and Zupanc (2019) [40] and Pajnič, Obal, and Zupanc (2020) [46]; Fattorini et al. (2019) [41] and Pajnič et al. (2022) [51]; Bleka et al. (2017b) [66] and Bleka et al. (2017a) [67]; and Turchi et al. (2019) [73] and Turchi et al. (2020) [77].

**Figure 4:**
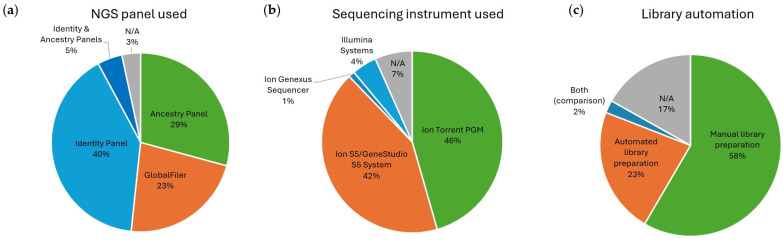
(**a**) Pie chart showing the proportion of articles using each NGS panel. N/A indicates articles for which the panel was not indicated. (**b**) Pie chart showing the sequencing instrument used. N/A indicates articles for which the instrument was not indicated. (**c**) Pie chart representing the proportion of articles employing manual rather than automated library preparation, or both. N/A indicates articles for which sequencing libraries were not prepared or the method used was not disclosed.

The data reported in Table 1 indicate that 20 articles focused on the use of the GlobalFiler NGS STR Panel, 36 exclusively on the Identity Panel, 26 on the Ancestry Panel, 4 used both Identity and Ancestry Panels, and 3 articles did not use any of these kits but considered the SNPs present in these panels (Figure 4a). The most commonly used instrument was the Ion Torrent PGM, employed in 41 publications, followed by the Ion S5/Ion GeneStudio S5 systems, used in 38 studies. The fully automated Ion Genexus Sequencer was used in one article, while the Illumina MiniSeq, MiSeq, and MiSeq FGx were used on four occasions (Figure 4b). Most of the publications relied on manual library preparation, while only 23% of the authors reported using automated library preparation, predominantly with the Ion Chef System (Figure 4c). Two articles performed a direct comparison between manual and automated library preparation, one of which also used the Biomek 3000 instrument. Seventeen percent of the articles either did not perform sequencing or did not clearly indicate whether automation during library preparation was used.

A review of the populations screened in these studies showed that, so far, more population data have been obtained from Asian ethnic groups, particularly Chinese, followed by some European populations, and only a few African and American groups (Table 2).

## 4. Discussion

### 4.1. Contributions by Country

Under the search criteria used in this review, 89 studies were identified covering the use of the Thermo Scientific Precision ID kits under consideration. The authors’ affiliations revealed contributions from 23 countries, with Europe as the leading continent, including Denmark and Italy as the highest contributors within Europe (Figure 2a). However, overall, China emerged as the country where most of the research concerning these kits was carried out, followed by the USA and Denmark. The 2020 survey on the implementation of MPS in European laboratories received wide response from German institutions (28.6% of all responses) due to the popularity of forensic DNA typing in the country’s police, academic, and private laboratories, followed by Italy with 8.6% and Spain with 6.7% [12]. However, while Italy and Spain both contributed to articles identified in this review, no contributions were found from German laboratories.

A scientometric study of the forensic science literature from 1975 to 2011 indicated the USA, UK, and Germany as the top three countries that contributed to the field [123]. A review of forensic genetics research over the last 40 years showed that the USA, China, Spain, Germany, UK, and Italy were the most productive countries [124]. It is perhaps surprising that no research using the Precision ID kits under consideration emerged with Germany and the UK as the primary author affiliations, apart from an article with combined Qatar/UK affiliations [109].

While the contribution by country may not fully reflect the number of working groups (whether publications come from the same or multiple institutions within a country), it does provide an overview of the global use of Thermo Fisher Scientific kits in research, highlighting the lack of outputs from countries that are typically very active in the field.

### 4.2. Contributions by Year

Since 2015, the number of publications rose to a yearly maximum of 17 in 2019 and has since stabilized at 7 to 11 publications per year (Figure 2b). This trend may suggest that the overall interest in using these kits for publishable research has surpassed its peak. However, as will be discussed further in this review, more research on the performance and population data associated with these panels is still needed. Given that over 100 to a maximum of 300 forensic genetics research articles have been published annually since 2007 [124], the yearly numbers highlighted in this review represent only a small percentage of all publications in the field.

### 4.3. Articles Type, Journal, and Access

This review highlighted that most of the publications were available only through subscription to the journal (Figure 2c). Open access journals allow research to be readily available to any reader, while showing comparable impact and quality to subscription journals [125]. In fact, two articles published in PLOS open access journals were among the most cited forensic genetics articles since 2022 [124]. However, most of the articles in this review were published in *Forensic Science International: Genetics* (FSI Genetics) (Figure 3a), which has restricted access, while the *Supplement Series* is openly accessible. Even though the *FSI Genetics* journal is not open access, it is accessible to members of the International Society for Forensic Genetics (ISFG), which includes more than a thousand members worldwide involved in the field [126].

The debate on the necessity of moving towards open-access only journals is currently active, with some advocating for it as the only way forward [127]. The fact that more than half the articles found in this review were published in *FSI Genetics* and the *Supplement Series* indicates that the ISFG journal and conference were the most popular venues for research concerning the Precision ID Panels. This was expected, as these journals have previously emerged as the most significant contributions to the forensic genetics field [124]. Although most articles were categorized as ‘Research Article’, the percentage of ‘Conference Article’ was relatively high (18%) (Figure 3b), indicating that various publications resulted from presentations at ISFG conferences. The review of the articles’ content (Table 1) also identified several publications where the subject matter was later developed into full research articles after initial publication in the *Supplement Series*. As these are often short articles presenting preliminary work, the number of fully developed research articles regarding the Precision ID Panels was reduced to 59.

### 4.4. Articles Focus and Current Knowledge

Out of all the articles, only 20 focused on the use of the GlobalFiler NGS STR Panel, while the majority were centered on the Identity Panel, followed by the Ancestry Panel (Figure 4a).

#### 4.4.1. Precision ID GlobalFiler NGS STR Panel

Almost all of the articles on the GlobalFiler NGS kit declared using the 35-plex NGS STR Panel v2. A preliminary version of the kit, including 32 markers (20 CODIS core loci, nine additional autosomal markers, plus a Y-chromosome STR, a Y InDel, and Amelogenin) was used in the first two articles published using the GlobalFiler NGS kit in 2017 and 2018. The first article was an evaluation of the kit on the Ion PGM System on Chinese Han individuals, showing full concordance across 31 loci typed with standard CE methods. Sensitivity was demonstrated down to 100 pg, with successful deconvolution of the minor contributor in 19:1 ratio mixtures. The study also achieved successful results from artificially degraded samples and case-type samples, including blood stains, muscle tissue, hair, semen stains, cigarette butts, and bone. This preliminary study showed promising results, indicating the kit’s reliability and robustness [35].

The second article provided a systematic evaluation of the kit along with the early-access Precision ID GlobalFiler Mixture ID Panel, which includes 113 markers (29 autosomal STRs, one Y-chromosome STR, 42 SNPs, two Y SNPs, 36 microhaplotypes, one Y InDel, and Amelogenin) [36]. An inter-laboratory evaluation on the Ion S5 System revealed the possibility of obtaining full profiles down to 62 pg of control DNA, aligning with the sensitivity previously observed for manually prepared libraries with the ForenSeq DNA Signature Prep Kit on the MiSeq FGx System [128]. The performance of the Precision ID GlobalFiler Mixture ID Panel was lower compared to the GlobalFiler NGS kit [36], with another study indicating similar performance in deconvoluting mixtures compared to the standard CE GlobalFiler kit [129]. Notably, the GlobalFiler Mixture ID Panel is no longer available for purchase on the Thermo Fisher Scientific website, suggesting that this has been discontinued [130].

The remaining 18 articles all used the Precision ID GlobalFiler NGS STR Panel v2. Several publications presented evaluations of the kit on the Ion S5 sequencers [38,54], with population data reported only for Japanese [48,49], Chinese [35,37], Indian (for microvariant alleles only) [47], and Spanish [43] population groups. In comparison, population data for STR sequence polymorphisms (and SNPs) contained in the ForenSeq DNA Signature Prep Kit have been generated for more populations, including French [131], Peruvian [132], Chinese [133], Danish [134], Mexican [135], and Qatari [136]. Furthermore, the Verogen kit has been used to generate sequence data for multiple global populations, including Caucasian, African, Asian, and American groups, to populate the STRSeq GenBank NCBI BioProject [21]. The availability of allele frequencies for global populations is fundamental for the application of these kits in forensic cases, as it allows for the calculation of statistical support. Efforts to populate databases and improve nomenclature and guidelines for the application of NGS STR kits are therefore vital to promoting their use in practice and are currently underway [6,21]. Even though the GlobalFiler NGS and ForenSeq kits share 24 autosomal and one Y-chromosome markers, different primer design developed by different manufacturers for the same loci can lead to null or shifted alleles and consequent discordances [137]. This highlights the need for investigating population data for different kits and systems, even though one study comparing shared loci from these two kits on different systems for 15 samples indicated concordance [54].

In general, these articles showed good performance of the GlobalFiler NGS kit, with high concordance with CE kits and increased match probabilities [35,43]. However, on rare occasions discrepancies were observed at loci D2S441 [48], Penta E [35,53], and Penta D [37,54]. The high interlocus imbalance and low depth coverage observed at Penta D appeared to cause inconsistent results for this locus, indicating the need for careful analysis [37,54]. Large imbalances at Penta D, leading to genotyping errors, were also reported for the ForenSeq kit on MiSeq FGx [138].

In terms of sensitivity, although the manufacturer suggests using a minimum of 125 pg of DNA, full profiles were reported with less than 100 pg, down to 62.5 pg [37] and 50 pg [54], and even with as little as 12 pg [38]. With full profiles achieved with 125 pg on the GlobalFiler CE kit [23] and 62 pg on the ForenSeq kit [128] and the Promega PowerSeq Auto System [139], the potential of the GlobalFiler NGS kit for low template samples appears significant. An optimal recovery PCR method was also reported for consistent production of full profiles with 50 pg of DNA [54].

Several articles explored the potential of the kit for mixture deconvolution. Ragazzo et al. (2020) showed that, compared to the GlobalFiler CE kit, the NGS kit was less powerful at detecting the minor contributor in mixtures of two individuals using urine and buccal cells. With buccal swabs, the minor contributor was deconvoluted at a 1:10 mixture ratio with CE and at 1:6 with NGS, while for urine, the deconvolution occurred at 1:20 with CE and 1:4 with NGS. However, the increased polymorphism achieved with sequence data led to higher discrimination power, partly due to the added information provided by the STRs’ flanking regions [44]. Full genotypes appeared easily attainable with 1:3 mixtures [37], while partial profiles were obtained even at an extreme 1:80 mixture ratio, although less reliable calls, more stutters, and artefacts were generated compared to CE testing [38]. The minor contributor was easily detectable in a 1:19 mixture not only with the GlobalFiler kit [35] but also with the ForenSeq [140] and PowerSeq Auto System [139]. The use of sequencing methods for mixture deconvolution was reported as beneficial due to the increased discrimination information, the potential for distinguishing stutters from alleles, the quantitative nature of reads, and the presence of SNPs in the STRs’ flanking regions [141,142]. Oldoni et al. (2019) showed better performance of NGS compared to CE for mixture deconvolution, thanks to the identification of peaks that would be masked by stutters with CE testing. In the same study, a 74-locus panel for microhaplotypes showed high potential for mixture deconvolution, with full profiles from the minor contributor attainable at 1:10 ratios and minimal dropouts at 1:20. Additionally, microhaplotypes profiles were also obtained from mixtures of three to five donors [42].

The performance of the kit with degraded samples was demonstrated in several studies. Full profiles were obtained from samples with a degradation index (DI) beyond 60 [54]. Considering that the ForenSeq kit on MiSeq FGx showed more than 90% dropout with DI beyond 72 [143], the result obtained with the GlobalFiler kit appeared promising. However, even with low DI, performance with bones and teeth samples appeared mixed, potentially due to the presence of inhibitors affecting the sequencing results and promoting dropouts [54]. The HID-Ion AmpliSeq Library Kit was tested on Ion PGM for common PCR inhibitors such as humic acid, melanin, hematin, collagen, and calcium, showing an inhibitory effect from humic acid, melanin, and collagen, and high inhibition from hematin. When the same inhibitors applied to samples tested with the ForenSeq kit on MiSeq FGx, greater inhibition was observed with melanin and collagen but resistance to hematin and calcium, highlighting different performances [33]. The GlobalFiler NGS kit was specifically tested for only one inhibitor, urea, obtaining full profiles only with concentrations ≤1000 ng/μL [37]. Bones and teeth from Second World War human remains were also tested in two publications from a Slovenian research group to support traditional CE STR testing in the identification of relatives, proving useful in improving statistical support [40,46]. A specific issue linked to analysis of degraded samples with NGS STR kits was highlighted as the generation of isometric artefacts, which are drop-ins that possess the same length as the parental allele with at least one nucleotide substitution. Although these have the potential to be interpreted as mixtures, their stochastic nature allows them to be identified through repeat testing, as they are not reproducible [41,51]. Insertion/deletion artefacts were previously highlighted as issues occurring with the Ion PGM [144], while sequencing with Illumina technology has been associated with substitution-type miscalls [145].

Apart from the Ion S5 System, the GlobalFiler kit was also tested on the fully automated Ion Torrent Genexus Sequencer, revealing sensitivity down to 100 pg, mixture deconvolution up to a 1:4 ratio, and suitability with degraded samples [50].

One publication highlighted the successful use of the GlobalFiler NGS kit, in addition to other STR kits, to help decipher a complex paternity case with three inconsistencies, allowing the identification of maternal uniparental disomy and confirmation of paternity [39]. This represents a strong example of the potential of NGS STRs in resolving complex paternity cases which are unresolved with standard CE [146]. Furthermore, the kit showed potential in differentiating monozygotic twins, identifying two SNPs in the flanking regions of two twin pairs out of 16 [52]. The ability to successfully discern between monozygotic twins remains a challenge in forensic science, with epigenetic analysis [147], rapidly mutating Y-chromosome STRs [148], and whole-genome sequencing (WGS) [149] all being explored for their potential in this context. Therefore, further investigation into the possibilities offered by STRs’ flanking regions may be warranted.

#### 4.4.2. Precision ID Identity Panel

The Precision ID Identity Panel was preceded by previous versions of the kit called HID-Ion AmpliSeq Identity Panel, the use of which was reported in articles published between 2015 and 2019. Articles using the Precision ID Identity Panel, on the other hand, were published between 2017 and 2023. As indicated in Table 1, 19 articles reported using the AmpliSeq panel, 4 of which indicated using version 2.2, while 2 used version 2.3, and 1 used version 4.0. The HID-Ion AmpliSeq Identity Panel, as well as the Precision ID Identity Panel, is composed of 124 SNPs, 90 autosomal markers for individual identification, and 34 Y-chromosome markers for male lineage investigation [57,62]. On the other hand, the HID-Ion AmpliSeq Identity Panel v2.2 included 169 SNPs and 136 autosomal (51 SNPforID and 85 Kidd Lab SNPs [27,28]) and 33 Y-chromosome markers [55,56], while v2.3, instead, was reported as having the same markers as the Precision ID kit [70]. Version 4.0 likely included the same number of markers, as it was reported as covering 289 SNPs when typed together with the HID-Ion AmpliSeq Ancestry Panel v4.0 [90]. Personal communication with Thermo Fisher Scientific customer support suggested that the AmpliSeq panels were the pre-official release versions. These were used for testing by some customers to guide in the definition of the final kit, which remained very similar. We believe this should be kept in mind when considering the published data for the kits, as some details on the performance of single markers may not necessarily reflect the exact performance of the final version of the kit.

All the HID-Ion AmpliSeq Identity Panel kits were sequenced on the Ion Torrent PGM instrument. Version 2.2 of the kit was tested and compared between three different laboratories, showing high coverage for most of the 169 SNPs down to 25–100 pg of DNA. However, uneven sequence coverage was observed for lower input DNA and discordant results were reported for five SNPs (rs1979255, rs1004357, rs938283, rs2032597, and rs2399332). Of these, rs938283 and rs1979255 are still included in the 124 Precision ID Panel [55]. Careful evaluation of the reliability of these markers, together with rs1029047 and another eight SNPs with inconsistent balance from a previous evaluation of the same 169-plex by Børsting et al. (2014) is prompted [55,150]. The study by Børsting et al. (2014) was not found among the articles to be reviewed when following the search parameters set in this literature review, as the name of the kit was indicated as Ion Torrent HID SNP and HID SNP primer panel v2.2 [150]. Rs1029047 was reported as giving inconsistent results in other NGS studies, likely due to misalignments caused by homopolymeric stretches flanking each side of the SNP [150,151,152,153]. Version 2.3 of the AmpliSeq kit was evaluated on a Han Chinese population sample, where markers rs214955, rs430046, rs7520386, rs876724, rs9171188, rs16981290, and rs2032631 showed poor allelic balance [70]. The same ethnic group was investigated in a kit evaluation that followed the Scientific Working Group on DNA Analysis Methods (SWGDAM) guidelines [154]. In this study, full profiles were obtained with 100 pg of DNA, full deconvolution of mixtures was achieved with a 1:9 ratio, and cross-reactivity with primates was observed. Rs430046, rs9866013, rs3182883, rs5746846, rs2567608, and rs4606077 were found to be imbalanced and were removed. However, overall, the kit revealed high discrimination power, robustness, reliability, and suitability for both individual identification and relationship testing [57].

While the Ion PGM was the only instrument used for the AmpliSeq kit evaluations, results for the Precision ID Panel were reported for both Ion PGM and Ion S5 Systems, as well as for the Illumina MiSeq and MiniSeq, with workflows adapted to allow typing [92,93]. An assessment of the Precision ID Identity Panel on Ion PGM, for example, showed good performance with forensic type samples, including blood, saliva swabs from objects, and sexual body fluids down to 200 pg of DNA. The same study showed full concordance between two different genotyping software, HID SNP Genotyper (v4.3.1) and CLC Genomics Workbench (Qiagen) [62]. Both Thermo Fisher Scientific with the HID SNP Genotyper and Illumina with the Universal Analysis Software (UAS) developed their own pipeline for analysis of data produced by their respective sequencers. Neither of these pipelines, however, allow easy visualization of the sequence alignment performed to generate the calls, which is liable to errors such as insertions and deletions. For this reason, a third-party workflow was developed in CLC Genomics Workbench as a tool to confirm allelic calls and compare genotypes. An initial study showed concordance of 99.8% between the manufacturer’s software and CLC, highlighting the need for careful evaluation and consideration of the software used [155]. An example of the importance of comparison between platforms and analysis methods emerged with the Apaga et al. (2017) study. Here, 83 shared markers between ForenSeq kit on MiSeq FGx and HID-Ion AmpliSeq Identity Panel on Ion PGM were compared, showing 99.7% concordance. Specifically, rs10776839, rs1031825, and rs1736442 appeared to require further evaluation, with almost 5% of discordances [32]. Li et al. (2023) also highlighted discordant genotypes at six SNPs among 83 shared markers between ForenSeq DNA Signature Prep Kit on MiSeq FGx, Precision ID Identity Panel on Ion S5 XL, and MGIEasy Signature Identification Library Prep Kit on MGISEQ-2000. The discordances were attributed to either primer binding region differences, misalignments, or insertion/deletion errors. Even if different performances were highlighted in terms of sequence quality, sequencing depth, allelic balance, and background noises, no method appeared better on all fronts. The Ion S5 XL was found to bear higher levels of allele-nonspecific miscalled reads and site- and genotype-dependent miscalled patterns, which deserve attention [87].

In addition to the two articles previously mentioned that typed the Han Chinese population, the Identity Panel markers were also screened in a Han Chinese group from the Hebei province. This study investigated forensic parameters, Y haplogroups, and 32 microhaplotypes observed in the targeted regions [71]. Tibetan, Uyghur, and Hui Chinese populations were also typed with the Identity Panel, revealing as many as 16 different Y haplogroups between different populations [69]. Apart from Chinese, population data were also obtained for Basques [64], Danes [59], Brazilians [74,75], Somali [63], Central Indians [83], Koreans [85], and Myanma people [86]. The study analyzing Koreans and Myanma individuals also investigated the microhaplotypes present on the flanking regions using the custom variant caller Visual SNP [156], which increased the kit discrimination power [85,86]. Data for Japanese and Malay populations were only reported in relation to the Y-chromosome SNPs included in the kit, reporting analytical issues related to multiple missing calls at rs2032599 and M479 and minor misreading at rs2032631 and M479 [58]. Overall, as per the GlobalFiler NGS kit, more research appears to be needed to improve the global population coverage and increase the reference data necessary for application to live cases. Since the discrimination power of the 90 autosomal SNPs contained in the Identity Panel was shown to match the discrimination power of 23 autosomal STRs, its application would prove useful in scenarios in which standard STR testing may not be applicable [85].

Excluding the AmpliSeq v2.2 kit previously mentioned, the other two publications that reported using this kit focused on mixture deconvolution. The LRmix software (v4.1), classically used for STR mixture interpretation, was proven successful in deconvoluting simple two-people mixtures (1:1, 1:3, and some 1:9) but failed when applied to three-people mixtures [56]. The performance of LRmix was also compared to a quantitative model for R called EuroForMix, which uses sequence read coverage to improve the deconvolution performance. With this method, the Likelihood Ratio (LR) of two-people mixtures was improved, also demonstrating advantages in the interpretation of three-people mixtures [66,67]. Eduardoff et al. (2015) showed the need for careful evaluation for the deconvolution of 1:9 mixtures [55]. However, identification of the presence of mixtures with a 1:100 ratio was achieved with NGS technology, underlining an advantage compared to CE methods, where the peaks would be difficult to distinguish from background noise [150,157]. Generally, SNPs are not ideal markers for mixture identification as they are more commonly bi-allelic and require evaluation of allelic imbalances as the only method to identify the presence of multiple contributors [150,157,158]. However, multi-allelic SNPs with high polymorphism are also available in the DNA and recent technologies allow screening of numerous markers simultaneously, increasing the performance for mixture analysis [158]. A Python script called SNPonPGM was also tested on HID-Ion AmpliSeq Identity Panel results, enabling identification of two-people mixtures up to a 1:24 ratio [59]. Another two methods for mixtures were assessed with the Precision ID kit, a Perl-based pipeline and R scripts. The Perl pipeline allowed good deconvolution of 1:1.5 and 1:4 mixtures, with lower performance for 1:4 and 1:9 mixtures and poor results for 1:19 mixtures [81]. The R algorithms appeared accurate for balanced mixtures and were only accurate for unbalanced mixtures when the minor contributor profile was known. Accurate estimation of the DNA mixtures was achieved up to a 1:6 ratio [84].

Beyond mixtures, a wide range of samples were tested with the Identity Panel kits. The AmpliSeq kit was proven successful in typing DNA extracted from buccal swabs samples on FTA cards [60], and case-type samples such as trace DNA, muscle, saliva, and blood stains, even when degraded. In fact, aged bloodstains with a DI of 1.76 and 3.44 revealed complete profiles with the NGS kit compared to the partial profiles obtained via CE testing with the PowerPlex Fusion STR System [57]. In this scenario, the improved performance of the NGS kit was likely due to the short amplicon length of the kit, between 75 and 242 bp, compared to a range of ~80–475 bp for the PowerPlex Fusion kit [57,159]. Informative profiles were also obtained when testing 1–1.2 ng of highly degraded DNA (DI > 9), with improved performance attained by using a consensus profile of 2–3 replicates and maintaining PCR cycles < 25–26 with a 50 reads coverage threshold to reduce the occurrence of sequencing errors [73,77]. Real forensic samples including semen, saliva, blood, and epithelial cells collected at crime scenes revealed higher statistical strength with the AmpliSeq Identity kit compared to PowerPlex Fusion STR testing. However, lower sequencing quality was shown for specific sample types, such as epithelial cells and saliva, which may require manual intervention during interpretation to avoid miscalls. This sample-specific variation was explained as possibly depending on environmental conditions such as contaminants and the presence of background DNA, more than the characteristics of the biological samples themselves [76]. Testing of human remains appeared challenging, with the AmpliSeq kit failing to sequence poor quality samples on the Ion PGM but proving useful in well-preserved samples [65]. On the other hand, the Precision ID Identity Panel in combination with the Ion Chef System allowed for the obtainment of full profiles from two Early Middle Ages skeletons to confirm relationship where standard STR testing was statistically too low [88]. Also, statistically acceptable results obtained from World War 2 remains appeared useful for profile confirmation, even if the presence of contamination led to the analysis being considered unreliable for exclusion purposes [89]. On top of this, both the Precision ID chemistry and the Signature Prep Kit demonstrated more successful results than traditional CE testing in generating profiles from bone and tooth samples exposed to DNA insults [160]. Other sample types that resulted in useful typing using the Identity Panel were DNA extracted from the gut of lice [61] and mosquitos [79]; fingerprints collected via columnar thin-film (CTF) deposition, a development method that showed no inhibition for NGS [78,82]; pre-natal DNA tests from maternal plasma/serum [68,72]; tumor tissue analysis to identify the body source [80]; and DNA obtained from touch and blood deposited on bomb fragments after detonation [91].

The benefits of using shorter amplicons to facilitate the amplification of degraded DNA and challenging samples is a concept that has long been considered by forensic scientists. For this reason, mini-STR kits with amplicon length < 200 bp were generated, even though these are still limited in multiplexing capabilities [161]. Amplicons for STR loci are restricted by the separation by size limitation of CE methods, as well as the length of the repeated region. On the other hand, SNPs are single nucleotide variants that can be tested maintaining short amplicons, also thanks to the multiplexing capabilities of NGS technologies. On top of this, their abundance allows for the generation of data with discrimination power that can match the STR assays, overcoming the intrinsic limited polymorphic value of SNPs and therefore providing advantages in the analysis of degraded samples [162].

Out of the articles reviewed, two did not involve direct sample processing with the kit but took the Identity Panel SNPs under consideration for comparisons. Huang et al. (2018) performed a genome-wide search to identify 117 universal SNPs with high minor allele frequency (>0.39) in 37 global populations and no associations (no linkage disequilibrium) [120]. When tested with this panel, a Chinese population sample revealed lower combined match probability compared to the Identity Panel results for the same number of markers on the same population, revealing an advantage in forensic scenarios [57,120]. However, the results for this panel were generated using the MassARRAY Genetic Analysis System, a custom array panel that uses mass spectrometry to detect SNPs [163]. Given that this is a completely different method compared to NGS, advantages and disadvantages and the possibility of using the same panel on sequencing platforms should be reviewed separately. Ragazzo et al. (2021) tested another genotyping method called OpenArray by creating a custom panel including 60 SNPs from the Identity Panel, the ForenSeq DNA Signature Prep Kit, and the phenotyping Hirisplex-s markers. This panel showed good concordance with expected results, high call rate down to 0.5 ng of DNA, and suitability with casework samples. Even if more in-depth validation studies are required to support this technology for forensic use, it could be suitable as the first line of typing in the investigative process. However, the low discrimination rate given by the reduced number of SNPs would likely still require further DNA testing for confirmation [121].

#### 4.4.3. Precision ID Ancestry Panel

Similarly to the Identity Panel, the Precision ID Ancestry Panel was preceded by the HID-Ion AmpliSeq Ancestry Panel, which was mentioned in five publications (Table 1). Two of these articles were introduced in the previous paragraph since the Ancestry kit was used together with the Identity kit. These were an evaluation of the kits on reference material on Ion PGM [90] and the analysis of DNA from bomb fragments [91]. The analysis of DNA from detonated bombs revealed better performance of insertions/null (INNUL) markers compared to CE and SNP sequencing, and acceptable performance of the ancestry kit for prediction, even if with low confidence results. The Precision ID Ancestry Panel was also used together with the Identity Panel on a workflow developed for Illumina MiniSeq [92] and in the evaluation of genotypes from dust samples [93].

In terms of sensitivity, the Precision ID Ancestry Panel on Ion PGM generated full profiles with 1 ng of DNA at 21 PCR cycles, and full profiles down to 30 pg when adopting 25 PCR cycles, indicating high sensitivity. However, even partial profiles obtained with 21 cycles with inputs down to 125 pg generated accurate biogeographical prediction with the manufacturer’s HID SNP Genotyper software. High-confidence predictions were also achieved down to 30 pg at 25 cycles. Continental level assignments were consistent with self-declared populations of origin, while the sub-population level assignments emerged as requiring further evaluation. Admixed samples, as well as degraded samples and mixtures, also appeared as necessitating more extensive work to improve accuracy [104]. In a separate study comparing manual and automated workflow on the Ion Chef System, full profiles were obtained from 120 pg of DNA, with no apparent difference between the two methods apart from the increased speed and reliability of automation [109]. Evaluation of the kit on Ion PGM showed consistently low performance of rs459920, rs7251928, and rs7722456 likely explained by misalignment of homopolymeric stretches [97]. The authors advised removing these loci from the analysis, prompting the need for further evaluations to confirm or withdraw the necessity for their exclusion and expand the assessment of the link between results, sample types, and the protocol and instrument used. The same authors reported increased interlocus balance variability compared to the Precision ID Identity Panel kit, which may constitute an issue of the presence of lower-quality samples [59,97].

The Ancestry Panel was tested not only on the Ion PGM but also on the Ion S5. A comparison study between the two platforms using forensic type samples was performed, indicating better performance of the Ion S5 in terms of speed, coverage, and SNP quality, even if both systems lead to concordant ancestry predictions [111]. The improved performance of the Ion S5 System compared to the Ion PGM was also reported for viral whole genome sequencing, leading to more high-quality reads at lower cost [164]. These differences indicate the need to take into account platform differences when evaluating panels’ performance.

In the context of ancestry predictions, the prediction algorithm/software used also deserves attention. One of the most popular methods for ancestry prediction analysis is the STRUCTURE software. This uses Bayes analysis to assign the most probable population to a profile through calculation of likelihood, which is based on combined genotype frequencies for each population [165]. STRUCTURE can use both SNPs and STRs to create clusters of populations with characteristic allelic frequencies, allowing prediction based on samples falling into the defined clusters. Principal Component Analysis (PCA) is also commonly used to summarize population data through multi-dimensional scaling (MDS), which reduces population variation to principal components representing the bulk of variation [166].

STRUCTURE was used, for example, to compare the predictive performance of almost 4000 individuals from 228 populations when using the Precision ID Ancestry Panel, rather than MAPlex or VISAGE Basic Tool panels. Similar results were obtained from all panels when using six broad continental regions as a parameter *K* set for the STRUCTURE analysis, which is the parameter commonly set for forensic investigations. When increasing *K* to 7 or 8, deviations from the expected results were observed, with the VISAGE Basic Tool set providing the most consistent results [122].

The Ion Torrent Suite software also performs ancestry assignments using either a population likelihood method or admixture prediction, the latter using seven root populations to assign the ancestral composition of the query. Jin et al. (2018) developed reporting guidelines to provide biogeographic ancestry services using this software (v5.2.0/v5.2.2), demonstrating the robustness of the kit on Ion S5 [102]. An open-source interactive platform called GenoGeographer was also recently developed to allow explorative analyses with graphical representations and statistical calculations. The software assigns the profile to the most likely population based on a likelihood ratio test that indicates the absolute concordance between a profile and the population. Z-scores indicating the deviation between observed and expected genotypes are calculated across each marker and combined to then calculate the *p*-value to support the given hypotheses [167]. The reference databases currently available on the software include Precision ID, Seldin loci, Kidd loci, and VISAGE Basic Tool for 41 global populations [168]. Precision ID Ancestry Panel profiles for 3603 individuals of known origins were used to generate reference population data for the GenoGeographer database. Testing of 566 extra profiles to determine z-score for ancestry prediction was performed, with 77.6% assigned. Of these, 83.6% were assigned to the expected population, with the remaining assignments being either ambiguous or discordant, indicating the need for more SNPs and/or more reference data to improve the prediction performance [110].

In this line, the GenoGeographer predictions obtained from the analysis of more than 1000 individuals from different countries were compared using data from both the Precision ID Ancestry Panel and a custom AmpliSeq EUROFORGEN NAME panel of 102 SNPs. Results showed a reduction in ambiguous assignment when combining the two panels, while indicating persistent limitations in separating Middle Eastern population groups from European and South-Central Asian populations [112]. Even if, overall, the method appeared useful, flaws were encountered with admixed populations. To attempt a reduction in the number of rejected results, an admixed ancestry module was added to the GenoGeographer test method so that profiles appearing as a combination of profiles from two populations could be recognized as such rather than being rejected [169].

The GenoGeographer software used on Precision ID Ancestry data from a heterogeneous population from Brazil led to 63% of profiles with no assigned population, which was reduced to 55% when the admixture option was used on failed samples. On top of this, an LR below 1000 emerged as a possible indicator of admixture [119]. Analysis using the GenoGeographer Admixture Module was performed on ~3500 individuals belonging to one of nine reference populations available on the website, indicating a reduction in rejection rate without increasing the error rate compared to using the standard test method. Overall, more than 95% of profiles were assigned to one or more populations, ~95% of which were concordant. The rejection rate was higher, however, for samples originating from populations not included in the reference database, especially for American samples [116]. Populations such as the Punjabi Pakistani analyzed with the Precision ID Ancestry Panel and GenoGeographer were identified as admixed and non-discernable from South Central and Middle Eastern population groups [108]. Limitations were also observed in distinguishing geographically proximate populations such as Turks and Iranians, who could not be differentiated, nor could they be separated from either European or South-Central Asian populations [96].

Apart from the GenoGeographer method, other investigations based on the Ancestry Panel such as the evaluation of genetic distances and the use of STRUCTURE were employed to evaluate heterogeneous samples from Brazilians, indicating the presence of admixture [118]. Overall, data for this kit were investigated for numerous populations, including Greenlanders [94], Brazilians [118,119], Basques [95], Turks [96,112], Iranians [96,112], Somali [97,112], Danes [97,112], Ecuadorians [98], Chinese ethnic groups [100,103,105,113,117], Koreans [105], Vietnamese [105], Nepalese [105], Indians [105], Pakistani ethnic groups [105,108,112,114], Japanese [101,105], Albanians [112,116], Greeks [112,116], Iraqi [112], Slovenians [112,116], Afghani [112], Eritreans [112,116], Moroccans [112], Portuguese [112], and Syrians [112].

The Ancestry Panel was found to be suitable not only for ancestry investigations but also for personal identification [100,103,117]. Results were also obtained from challenging samples, including carbonized corpse [99], skeletal remains via MiSeq FGx sequencing [107], and touch samples recovered from multiple surfaces and typed via direct PCR [106,115]. Direct PCR was demonstrated as an effective method to obtain STR profiles from low-quantity and -quality DNA samples, including touch samples. This is due to the reduced DNA loss that is usually encountered when performing DNA extraction and quantification, as well as a reduction in DNA contamination chances and in processing time [170]. Young et al. (2019) showed, for the first time, the possibility of performing direct PCR on latent DNA using MPS with the QIAGEN 140-SNP forensic identification multiplex kit [171]. The same research team showed a higher detection rate with the Precision ID kit compared to the QIAGEN kit, with a similar concordance rate [106]. In a separate study, full profiles were obtained from 23% of touch samples using the Ancestry kit on the Ion GeneStudio S5 Plus instrument, with partial profiles attained from 32% of the samples. Additionally, correct ancestry was assigned to 90% of the samples when at least 70% of the SNPs were called [115]. Differently from what shown by Al-Asfi et al. (2018) using the Ion PGM instrument, these results were obtained without the need to increase PCR cycles [104,115].

### 4.5. Sequencing Instrument and Automation

The current review highlighted that the Ion Torrent PGM System was employed in more publications compared to the Ion S5/Ion GeneStudio S5 Systems, with only one publication using the fully automated Ion Genexus Sequencer and four using Illumina sequencers (Figure 4b). As indicated in Table 1, on three occasions, the instrument used was not disclosed. Currently, Thermo Fisher Scientific sells the Ion GeneStudio S5 Systems, the Genexus Systems, and the Ion PGM Dx System (which differs from the Ion PGM), while the other instruments appear discontinued [172]. Given the continuous improvements of the instrument manufactured and the different performance reported between platforms [63,87,111,164,173], it is crucial to ensure that new validations and studies are conducted to keep up with the latest technologies and maintain the high performance levels of the kits.

The utilization of manual versus automated library preparation also emerged as a source of differences [36,63], with most of the research groups still relying on manual library preparation (Figure 4c), which, on one occasion, was shown to achieve higher sensitivity compared to the automated method [36]. Because of these differences, when assessing and comparing the performance of sequencing kits, methodological differences including library preparation method should be taken into consideration. However, we observed that in several articles, this information was not disclosed.

### 4.6. Limitations

This review study encountered several limitations. The number of working groups that contributed to the research surrounding the Thermo Fisher Scientific kits under consideration was not established. This would have further helped to determine the popularity of the kits, as a number of publications were produced by the same researchers. Also, several articles were revealed to constitute expansions of the same research study, repurposed from streamlined publications, e.g., conference articles, to more developed research articles. This factor also influenced the evaluation of the most-used sequencers and sequencing methods, as well as the number of publications per population of origin.

Moreover, it is worth noting that the popularity of kits and instruments in the practice, as highlighted in previous surveys [11,12], is not necessarily reflected in published research. Even though the findings from the surveys appeared to align with the results of this review, it is possible that more private and public laboratories not involved in research may be using one method more prevalently than another.

Limitations were also identified in the variables that may affect the comparison of different studies when assessing the kits’ performance. As different authors used different extraction, quantitation, and library preparation methods, as well as different instruments, it was hard to evaluate whether articles’ findings were linked to these variables rather than associated with the kits’ characteristics. Furthermore, the lack of available information regarding the differences between kits and instruments that undergo name changes can also affect the generalization of findings. It is unclear, for example, if the differences between the various versions of HID-Ion AmpliSeq kits encountered and the final Precision ID kits would be sufficient to affect the findings. Similarly, it is unclear whether the Ion S5 and Ion GeneStudio S5 Systems should be considered as different instruments.

Lastly, since NGS errors may be associated not only with experimental conditions, but also with sample characteristics, PCR bias, analysis procedures, and software used [76], the evaluation of kits performance should take these variables into consideration. Results may be skewed, for example, when using different software and software versions. Müller et al. (2018), for example, showed differences between the noise artefacts detected between Converge version 1 and 2 [36], which were not further discussed in this review. This underscores the importance of always clearly defining all materials and methods used in investigations.

### 4.7. Future Perspectives

Starting from the discussed limitations, further studies may help clarify the differences between methods and kits and isolate the multiple identified variables. Further work should be undertaken to evaluate SNPs with high imbalance, to either improve their performance or establish the need for their removal. This should also be assessed in relation to the instrument and software used. Additional studies to evaluate the sensitivity of manual rather than automated library preparation methods should be considered, as this could affect the methodology of choice for the introduction of NGS into routine use for challenging casework samples. Efforts should be placed into increasing the performance of automated methods and promoting their use in the practice, as automation delivers several important advantages. These include faster processing time, increased robustness and reproducibility, reduced opportunities for human error, and lower contamination rate [174].

More research could also explore the different effects of sample characteristics and extraction methods for different instruments, with particular attention to the software used and the effect on artefacts and interlocus imbalance. The research mentioned in this review indicated no overall better instrument when comparing different sequencing quality parameters for the Ion Torrent and the Illumina platforms with their corresponding forensic kits [32,87]. However, a review of the ForenSeq DNA Signature Prep Kit research could be beneficial for comparison with the present study.

Beyond the methodological evaluations, more research is needed to fully assess the added value of NGS analysis in mixture deconvolution compared to CE testing. This is due to several studies reporting that standard STR testing performs better, with others, on the other hand, indicating advantages specific to sequencing technologies. Limited knowledge regarding the effect of inhibitors on the kits under consideration and the relative platforms were also observed, which warrants further investigation.

Another promising area of study is the possibility of differentiating twins by using STR flanking region information, as shown in the preliminary study by Fonseca and Fridman (2022) [52]. Further research into the presence of microhaplotypes in the flanking regions of the Precision ID kit could also provide valuable data for multiple applications.

More studies are also needed to analyze and produce population data for the Precision ID kits, particularly for the GlobalFiler kit, where information on the expected alleles specific for the primers used is currently limited but fundamental to understand the kit behavior in each population. In fact, lack of population data to back statistical support calculations is considered among the top four factors limiting the implementation of NGS in forensic laboratories. The other main challenges are represented by lack of consistent nomenclature and standards for reporting, lack of compatibility with existing databases, and lack of a developed legislative framework regarding the use of data that may have health implications [11,175,176]. Additionally, the current lack of training workshops and proficiency testing to ensure that laboratories can work effectively, compare results, and achieve accreditation is a primary aspect that deserves further attention and development.

### 4.8. Ethical Considerations

Last but not least, ethical considerations must remain at the forefront of future research to ensure that all human subjects and the ethnicities they represent are respected and protected. Appropriate ethical approval must be obtained from the relevant review boards and disclosed in the studies, while informed consent must be obtained from all individuals involved in the research. Personal data and the genetic information collected must be protected, anonymizing participants and reducing data sharing only to the researchers directly involved in the study that necessitate access. The studies must be conducted ethically, with transparency and honesty, and taking into consideration the potential impact of findings involving genetic traits on the relative populations. Most importantly, the research should benefit society and contribute to the advancement of forensic genetics while minimizing harm to participants and communities.

Unfortunately, in recent years, concerns have been raised about forensic genetics investigations not adhering to some of these fundamental ethical principles. More specifically, ethical issues regarding systematic collection of DNA from oppressed minorities through law enforcement and failures in obtaining genuine informed consent were brought to light. These concerns involved mainly Chinese ethnic minorities, especially Uyghur and Tibetan people, whose DNA may have been collected unethically and for unethical purposes, including biometric analysis for discriminative facial recognition systems. The potential for abuse appeared more obvious in Xinjiang Uyghur Autonomous Region, where increasing evidence of oppression of Muslim minorities emerged [177,178]. In the present review, China was the most productive country and the country that published data from the highest number of ethnic minorities, including Han, Hui, Tibetan, and Uyghur (Table 2). Liu et al., 2018 [69] and He et al., 2018 [103] both declared using data collected from Uyghur individuals residing in the Xinjiang Uyghur Autonomous Region. Also, during the article selection process, a retracted article entitled ‘*Analysis of Uyghur and Kazakh populations using the Precision ID Ancestry Panel*’ was encountered, followed by an editorial expression of concern regarding ethical issues [179,180]. While the retraction indicates active efforts to enforce ethical requisites, there is an increased need for careful review and evaluation of the moral aspects of research methods, not only for new publications, but also for already published material.

The ISFG has recently established a Forensic Databases Advisory Board (FDAB) to provide recommendations on ethical considerations for forensic genetic frequency databases. However, its current focus is primarily on the Y-chromosome Haplotype Reference Database (YHRD), the EDNAP Mitochondrial DNA Population Database (EMPOP), and the STRs for Identity ENFSI Reference Database (STRidER) [181]. Little reference is made in their first report to sequencing methods and databases, which deserve particular attention given the extent of information that can be gathered with these technologies, including phenotypical and health data. On the other hand, certain countries are making active efforts to protect, for example, susceptible indigenous communities in genomic research, with frameworks being developed [182,183]. While gathering population data is fundamental to support statistical calculations for the appropriate analysis of forensic evidence, it remains paramount that studies are conducted with the highest level of integrity.

## 5. Conclusions

Forensic genetics is currently dominated by the well-established STR testing via capillary electrophoresis method. However, the advantages provided by new technologies, such as NGS, are leading researchers to dedicate part of their efforts to validation and investigation of these newer methods. Thermo Fisher Scientific is one of the most popular manufacturers in the field, producing, among others, the Precision ID GlobalFiler NGS STR, Identity, and Ancestry Panels. This review aimed at investigating the state of the art of the research surrounding these kits, focusing on their popularity, applications, and performance.

This review highlighted an uneven distribution of research across countries, with China being the most productive in the field. In line with this, most of the population data obtained for these kits was produced for Asiatic countries, for some of which some ethical concerns have unfortunately been raised. More data for populations of other continents are still required to increase database sizes and promote NGS use. Additionally, greater efforts to produce open-access research are necessary to ensure the widest distribution of findings.

The Precision ID GlobalFiler NGS STR Panel appears to be a solid kit, useful for individual identification, complex paternity cases, studies of human remains, and with potential in twin differentiation. Excluding the presence of isometric artefacts and a few issues encountered with certain problematic loci such as Penta D and Penta E, the kit revealed good performance and high concordance. The sensitivity was shown to be particularly high, with numerous accounts of successful results with less than 100 pg and a report of successful full typing with as little as 12 pg. Mixed results were observed in the presence of mixtures, with different results reported by different authors and differences detected according to the sample type. On top of this, generally better results were obtained with standard CE, even if more discrimination power was achieved with the Precision ID kit. The use of the kit on challenging samples, on the other hand, appeared useful to deliver added data to support STR testing and increase statistical support, depending, however, on the overall quality of the sample. Apart from urea, inhibition studies were found to be lacking. More efforts are also needed to increase the population data available, as only data for four populations using this kit have been published.

The Identity Panel emerged as a popular kit, with high sensitivity (beyond 100 pg) and high concordance with expected results. However, some instances of markers with uneven coverage were highlighted, especially in preliminary versions of the kit. The software used to assign the allelic calls also transpired as a possible source of variability, which deserves attention. With its 34 Y-chromosome markers, this panel also came to light as a useful kit for Y chromosome investigations, including identification of Y haplogroups for population studies. However, population data generated with the kit should be expanded to enrich reference databases. The kit also emerged as a useful tool for mixture identification, with several software products developed for this purpose. Reports of identification of 1:100 mixtures and partial success with three-people mixture deconvolutions were all promising findings. Additionally, the kit performed better with challenging and degraded samples compared to STR CE testing, with profiles obtained from insect guts, fingerprints, biological stains on detonated bombs, and pre-natal DNA, demonstrating its utility in multiple applications.

The Ancestry Panel also showed very good sensitivity, with full profiles obtained from only 30 pg when PCR cycles were increased from 21 to 25. However, some loci with high interlocus imbalance were identified, requiring further evaluation. Despite this, good biogeographical ancestry predictions were often achieved, even with partial profiles. Regardless of the several available methods for ancestry prediction, some common limitations were highlighted. These included limited prediction efficiency when dealing with mixtures, degraded samples, admixed samples, subpopulations, and populations lacking a comprehensive reference database. The expansion of reference data and an increase of SNPs in the panel should therefore be considered to work towards more accurate predictive methods. Besides ancestry, the kit was proven useful for individual identification, providing enough statistical support to substitute STR kits with the advantage of increased suitability for challenging samples. The success shown with the adoption of direct PCR on latent DNA opens further possibilities for applying the kit in forensic cases.

Despite the promising results, more efforts are still needed, both in and out of the laboratory, to fully support the introduction of these kits in forensic settings for use in live cases. However, with the expansion of databases and greater efforts to define appropriate guidelines and frameworks for the use of next-generation sequencing in forensics, we believe that the time for its introduction is approaching. In this context, we hope that this review provides the necessary background for laboratories considering working with these Precision ID kits, suggesting areas of attention, further research, and improvement.

## Figures and Tables

**Table 1:** List of articles included in this review, with details on the sequencing panel and instrument used for the processing. The articles are presented in chronological order within four sections defined by the panel used (GlobalFiler NGS STR kit, Identity Panel, Identity and Ancestry Panel together, and Ancestry Panel). N/A was used when the information was not applicable or not disclosed in the original publication. A summary of the main points of each article is also reported.

Reference	Sequencing Panel	Sequencing Instrument	Main Points
Wang et al., 2017 [35]	Precision ID GlobalFiler NGS STR Panel	Ion Torrent PGM	Evaluation of the kit on Ion PGM. Population data for Chinese Han were obtained. Minor contributor of mixture could be detected in 19:1 mixture. Good results were obtained from multiple casework-type samples.
Müller et al., 2018 [36]	Early Access Precision ID GlobalFiler NGS STR Panel	Ion S5 System	Inter-laboratory evaluation (two laboratories) of prototype kits, Early Access Precision ID GlobalFiler Mixture ID and GlobalFiler NGS STR Panel. Higher sensitivity was revealed with manual library preparation (full profiles at 62 pg) compared to automated.
Tao et al., 2019 [37]	Precision ID GlobalFiler NGS STR Panel v2	Ion S5 System	Evaluation of the kit on Ion S5. Population data for Uyghur (inner Asia) were obtained. Full profiles were generated down to 62.5 pg and from 1:3 mixtures. Discordances with GlobalFiler CE kits were observed at Penta D due to high interlocus imbalance. Urea concentrations higher than 1000 ng/μL inhibited profiling.
Faccinetto et al., 2019 [38]	Precision ID GlobalFiler NGS STR Panel v2	Ion GeneStudio S5 System	Internal validation of the kit. Complete profiles were obtained with 12 pg of DNA. Partial profiles were generated from 1:80 mixtures. More stutters and noise artefacts were observed in the NGS kit compared to CE with the GlobalFiler kit, which also produced more reliable calls from mixtures.
Shyla et al., 2019 [39]	Precision ID GlobalFiler NGS STR Panel v2	HID Ion GeneStudio S5 System	Application of the kit for a complex paternity case with three incompatibilities. Uniparental disomy from the mother was proven.
Pajnič, Pogorelc and Zupanc, 2019 [40]	Precision ID GlobalFiler NGS STR Panel (v2)	Ion S5 System	Human remains from World War 2 were processed for kinship analysis, on top of ESI 17 and NGM kits STR typing. The added information obtained with the GlobalFiler NGS kit proved useful.
Fattorini et al., 2019 [41]	Precision ID GlobalFiler NGS STR Panel v2	Ion S5 System	Isometric artefacts were identified from analysis of heat-degraded samples. These should be evaluated carefully as they could be interpreted as mixtures.
Oldoni et al., 2019 [42]	Precision ID GlobalFiler NGS STR Panel v2	Ion S5 System	A microhaplotype panel for Ion S5 was compared to GlobalFiler NGS and CE kits for mixture deconvolution. The GlobalFiler NGS kit performed better than CE for mixtures, and the microhaplotype panel performed even better, with 50 pg sensitivity and full profiles attainable from three–five people mixtures.
Barrio et al., 2019 [43]	Precision ID GlobalFiler NGS STR Panel v2	Ion S5 XL System	Spanish population data were reported. Comparison of the GlobalFiler NGS results with the PowerPlex Fusion 6C STR kit showed high concordance. Increased discrimination power was obtained with sequence data.
Ragazzo et al., 2020 [44]	Precision ID GlobalFiler NGS STR Panel v2	Ion S5 System	Evaluation of the kit for mixture interpretation from saliva and urine samples. The GlobalFiler CE kit performed better at detecting the minor contributor, but the NGS kit provided increased discrimination.
Wang et al., 2020 [45]	Precision ID GlobalFiler NGS STR Panel v2	Ion S5 XL System	Chinese Han and Tibetan population data were reported. Increased variation was revealed from sequence data compared to CE data. Discordances at Penta E and interlocus imbalance were highlighted.
Pajnič, Obal and Zupanc, 2020 [46]	Precision ID GlobalFiler NGS STR Panel v2	Ion S5 System	Human remains from World War 2 were processed for kinship analysis, on top of autosomal STR and Y-STR kits. The added information obtained with the GlobalFiler NGS kit proved useful to increase posterior probability.
Dash et al., 2021 [47]	Precision ID GlobalFiler NGS STR Panel v2	Ion S5 System	Central Indian population samples were evaluated for microvariant alleles, which appeared to be rare.
Kitayama et al., 2022 [48]	Precision ID GlobalFiler NGS STR Panel v2	Ion S5 XL System	Japanese population data were reported. Discrepancy at locus D2S441 was observed with CE data from the GlobalFiler STR kit.
Ohuchi et al., 2022 [49]	Precision ID GlobalFiler NGS STR Panel v2	Ion S5 System	Japanese population data were obtained for database generation. Care in evaluation of sequence errors and stutters was advised.
Guo et al., 2022 [50]	Precision ID GlobalFiler NGS STR Panel v2	Ion Genexus Sequencer	Evaluation of the kit on Ion Torrent Genexus, a new automated technology for library preparation and sequencing coupled with analysis software for quick and easy profile generation. Sensitivity down to 100 pg of DNA was shown, while the minor contributor was identified in a 1:4 mixture.
Pajnič et al., 2022 [51]	Precision ID GlobalFiler NGS STR Panel v2	Ion S5 System	Isometric artefacts were identified from analysis of heat-degraded samples. Additionally, 5.9 artefacts per test were observed, 5.2% with higher coverage than the original allele. These artefacts are stochastic and represent a particular issue with Ion Torrent technology.
Fonseca and Fridman, 2022 [52]	Precision ID GlobalFiler NGS STR Panel v2	Ion GeneStudio S5 System	The kit was used to analyze sequence variation between monozygotic twins for differentiation purposes. Two SNPs were identified in the flanking regions, which highlights the possibility of using this kit to differentiate identical twins.
Kocsis, Matrai and Egyed, 2023 [53]	Precision ID GlobalFiler NGS STR Panel v2	Ion S5 System	Evaluation of discordances at locus Penta E between the GlobalFiler NGS STR kit, PowerPlex Fusion 6C, and PowerPlex 18D Systems CE kits. Sanger sequencing was performed.
Sharma and Wurmbach, 2024 [54]	Precision ID GlobalFiler NGS STR Panel v2	Ion S5 System	Kit evaluation on Ion S5. Optimal recovery PCR method was used. Full profiles were obtained from 50 pg of DNA, and degraded samples with Degradation Index (DI) > 60. 1:30–1:20 mixtures were identified using read count. High locus imbalance was observed at locus Penta D. Most artefacts were detected at D12S391.
Eduardoff et al., 2015 [55]	HID-Ion AmpliSeq Identity Panel v2.2	Ion Torrent PGM	Evaluation of the Identity kit between three laboratories; 25–100 pg of DNA were found to deliver acceptable results.
Gill et al., 2015 [56]	HID-Ion AmpliSeq Identity Panel v2.2	Ion Torrent PGM	The LRmix program was used for deconvolution of two–three people mixtures. Two people mixtures gave good results, while three people cases had variable success.
Guo et al., 2016 [57]	HID-Ion AmpliSeq Identity Panel	Ion Torrent PGM	Evaluation of the kit following the Scientific Working Group on DNA Analysis Methods (SWGDAM) guidelines. Data for Han Chinese were obtained. Cross-reactivity with primates was observed. Full deconvolution was obtained with 1:9 mixtures.
Ochiai et al., 2016 [58]	HID-Ion AmpliSeq Identity Panel	Ion Torrent PGM	Only Y-chromosome SNPs were considered. Data for Japanese and Malay were produced. Missing results and minor misreading were highlighted.
Buchard et al., 2016 [59]	HID-Ion AmpliSeq Identity Panel	Ion Torrent PGM	The panel was validated in an accredited laboratory for relationship testing. Thresholds were set and a Python script for analysis was devised. A 1:24 mixture of two people was identified by the script.
Kampmann et al., 2016 [60]	HID-Ion AmpliSeq Identity Panel	Ion Torrent PGM	Buccal swab samples on FTA cards were successfully processed.
Pilli et al., 2016 [61]	HID-Ion AmpliSeq Identity Panel v2.3	Ion Torrent PGM	Human DNA was extracted and successfully typed from lice for both STRs (AmpFℓSTR NGM Select kit) and SNPs (Identity Panel). More successful results were obtained with NGS SNP testing.
Meiklejohn and Robertson, 2017 [62]	Precision ID Identity Panel	Ion Torrent PGM	Evaluation of the kit on Ion PGM. Two types of genotyping analysis software were used, the Ion Torrent HID SNP Genotyper and CLC Genomics Workbench, achieving 100% concordance.
van der Heijden et al., 2017 [63]	Precision ID Identity Panel	Ion Torrent PGM and Ion S5 System	Comparison between manual library preparation with Ion PGM sequencing, automated library preparation (Biomek 3000) with Ion PGM sequencing, and automated library preparation (Ion Chef) with Ion S5 sequencing. The Ion Chef/Ion S5 workflow gave the best results but was more expensive. Somali population data were generated.
Garcia, Soto and Yurrebaso, 2017 [64]	HID-Ion AmpliSeq Identity Panel	Ion Torrent PGM	Forensic evaluation of the kit on Ion PGM. Population data for Basques were obtained.
Juras et al., 2017 [65]	HID-Ion AmpliSeq Identity Panel	Ion Torrent PGM	Human remains from neolithic graves were processed for kinship analysis. Illumina mtDNA sequencing was also performed. The SNP NGS kit was inefficient with poorly preserved ancient samples and was only useful with well-preserved samples.
Apaga et al., 2017 [32]	HID-Ion AmpliSeq Identity Panel	Ion Torrent PGM	Comparison of shared markers between ForenSeq kit on MiSeq and Identity Panel on Ion PGM. Different performances were highlighted. Some SNPs with observed discordances require attention.
Bleka et al., 2017b [66]	HID-Ion AmpliSeq Identity Panel v2.2	Ion Torrent PGM	Two–three people mixtures were analyzed to evaluate deconvolution performance of the quantitative model EuroForMix compared to the qualitative model LRmix. The discrimination power appeared improved.
Bleka et al., 2017a [67]	HID-Ion AmpliSeq Identity Panel v2.2	Ion Torrent PGM	Two–three people mixtures were analyzed to evaluate the deconvolution performance of the quantitative model EuroForMix compared to the qualitative model LRmix. The discrimination power appeared improved.
Cho et al., 2017 [68]	HID-Ion AmpliSeq Identity Panel	Ion Torrent PGM	DNA was successfully typed from cell-free DNA from pregnant mothers’ serum as a method to perform pre-natal DNA test.
Liu et al., 2018 [69]	Precision ID Identity Panel	Ion Torrent PGM	Tibetan, Uyghur, and Hui population data were generated. Y haplogroups were compared with worldwide populations.
Li et al., 2018 [70]	HID-Ion AmpliSeq Identity Panel v2.3	Ion Torrent PGM	Southern Chinese (Han) population data and forensic parameters were evaluated. Poor allelic balance was shown for seven markers, and 0.82% of miscalled reads were found. Y haplogroups were also investigated.
Sun et al., 2019 [71]	Precision ID Identity Panel	Ion Torrent PGM	Chinese Han (Hebei province) population data and forensic parameters were evaluated. Y haplogroups were investigated and microhaplotypes identified.
Christiansen et al., 2019 [72]	Precision ID Identity Panel	Ion S5 System	Paternal SNPs were successfully typed from cell-free DNA from pregnant mothers’ plasma to perform pre-natal DNA test. False calls were found, indicating the need for at least duplicate testing as these false calls were not reproducible. Paternal SNP dropouts could be reduced by using smaller amplicons.
Turchi et al., 2019 [73]	Precision ID Identity Panel	Ion Torrent PGM	The panel was successful with challenging samples and degraded and low-quantity DNA. Informative profiles were obtained from 1.2 to 1 ng of DNA with DI > 9. Most challenging samples could benefit from the generation of consensus profile of two to three replicates, from using <25–26 PCR cycles, and applying 50 reads coverage threshold.
Bottino, Silva and Moura-Neto, 2019 [74]	HID-Ion AmpliSeq Identity Panel	Ion Torrent PGM	The article reports forensic evaluation and investigation of Y haplogroups for Brazilians. Only twelve individuals were typed, but they appeared to represent the expectations for the general Brazilian population.
Avila et al., 2019b [75]	HID-Ion AmpliSeq Identity Panel	Ion Torrent PGM	Forensic evaluation and population data for Brazilians were produced. Y haplogroups were also investigated. The kit appeared suitable for both identity and kinship testing. Comparison with worldwide population was performed. Improvements in inter- and intralocus balance were highlighted as required.
Avila et al., 2019a [76]	HID-Ion AmpliSeq Identity Panel	Ion Torrent PGM	Brazilian casework samples were processed with the PowerPlex Fusion STR kit and Identity Panel SNP kit. The statistical strength of NGS results was higher than STRs. Epithelial cells generated lower-quality results. Manual review of NGS data appeared fundamental.
Turchi et al., 2020 [77]	Precision ID Identity Panel	Ion Torrent PGM	The kit was successful with challenging samples and degraded and low-quantity DNA. Good results were obtained down to 12 pg of degraded DNA. The most challenging samples could benefit from three replicates testing and 50 reads coverage threshold.
Tiedge et al., 2020 [78]	Precision ID Identity Panel	Ion S5 System	PowerPlex Fusion 6C STRs and Identity Panel SNPs were typed from prints developed using the columnar thin-film (CTF) method. No inhibition from CTF was shown for both methods, but more information was obtained from the NGS kit for low-quality/quantity DNA. A maximum of 27 PCR cycles was recommended.
Gray et al., 2020 [79]	Precision ID Identity Panel	Ion S5 System	STR (PowerPlex Fusion 6C) and SNP (Identity Panel) profiles were obtained from mosquitos after feeding on blood. All SNPs were obtained after 48 h from feeding (single source) or 24 h (mixtures), while all STRs were obtained after 24 h (single source) or 20 h (mixtures). However, STR was more efficient for mixture interpretation.
Sun et al., 2020 [80]	Precision ID Identity Panel	Ion Torrent PGM	Tumor tissue was profiled to identify the body source. More than 99% accuracy was obtained using 69–89 threshold when counting the number of loci with two alleles shared.
Chen et al., 2020 [81]	Precision ID Identity Panel	Ion Torrent PGM	A Perl-based pipeline was developed to deconvolute mixtures of two people. The method worked well even when mixed samples were first-degree relatives. The 1:19 mixtures showed poor results compared to 1:4 and 1:9, which were also worse than 1:1.5 and 1:4 results.
Tiedge et al., 2021 [82]	Precision ID Identity Panel	Ion S5 System	Fingerprints exposed to different environmental conditions and collected via CTF were typed. All samples yielded complete profiles and no inhibition from CTF was shown; 23 PCR cycles were used with 0.5–1 ng DNA and 29 for DNA input < 0.5 ng.
Dash et al., 2022 [83]	Precision ID Identity Panel	Ion GeneStudio S5 System	Central Indian population data were produced. Y-chromosome SNPs were investigated and interpopulation comparison was performed.
Yin, Zhang and Xing, 2022 [84]	Precision ID Identity Panel	HID Ion GeneStudio S5 System	R algorithm was devised and tested for mixture deconvolution. The deconvolution accuracy was high for balanced mixture ratios or unbalanced with known minor contributor, and the ratio was estimated correctly from 1:1 to 1:6 ranges.
Yang, Lee and Lee, 2023 [85]	Precision ID Identity Panel	HID Ion S5/Ion GeneStudio S5	Korean population data were produced and compared with other populations. Visual SNP caller was used to obtain microhaplotypes. The use of 16 microhaplotypes improved the discrimination power. Y-haplogroups were investigated. Population databases for the use of this kit for forensic application are still lacking.
Joo et al., 2023 [86]	Precision ID Identity Panel	Illumina MiSeq System	Myanmar population data were produced and compared with other populations. The kit was used on the MiSeq instrument. Microhaplotypes were found using Visual SNP caller.
Li et al., 2023 [87]	Precision ID Identity Panel	Ion S5 XL System	ForenSeq DNA Signature Prep Kit on MiSeq FGx, Precision ID Identity Panel on Ion S5 XL, and MGIEasy Signature Identification Library Prep Kit on MGISEQ-2000 were sequenced and compared. All performed differently, with no platform performing consistently better for all parameters considered. Six SNPs had discordant calls.
Pajnič, Leskovar and Cresnar, 2023 [88]	Precision ID Identity Panel	Ion GeneStudio S5 System	The Identity Panel was used together with the Investigator EssplexPlus SE QS kit for full siblings’ prediction from Early Middle Ages skeletons. The prediction was inconclusive with STRs only but successful with SNPs.
Fattorini et al., 2023 [89]	Precision ID Identity Panel	Ion S5 System	World War 2 bone remains delivering no results on STR testing were processed with the Identity Panel. The average DNA quantity was 6.8 pg; 93.8% of libraries produced results for 63/90 autosomal markers; 40% of results did not match the donor or were mixed profiles, possibly due to contamination.
Kiesler, Gettings and Vallone, 2015 [90]	HID-Ion AmpliSeq Identity Panel v4.0 and Ancestry Panel v4.0	Ion Torrent PGM	Evaluation of Identity and Ancestry Panels on NIST samples for the establishment of reference material. Some markers (0.25% for Identity and 2.02% for Ancestry Panel) showed coverage below recommended values of 300 X. Some markers in both panels showed allelic imbalance and strand bias (ratio of positive strand reads to negative strand reads). Discordant replicates were observed on two occasions in the Ancestry Panel.
Tasker et al., 2017 [91]	HID-Ion AmpliSeq Identity Panel and Ancestry Panel	Ion Torrent PGM	DNA was typed from touch/blood samples recovered from bomb fragments for ancestry inference. The InnoTyper 21 Kit for insertions/null markers was also used and appeared good for challenging samples. The success of the SNP NGS kits was variable.
Scheible et al., 2021 [92]	Precision ID Ancestry and Identity Panels	Illumina MiniSeq System	Workflow for Ancestry and Identity Panels was tested on Illumina MiniSeq; 93.9% of SNPs were successfully genotyped from positive controls, buccal swabs, and dust samples.
Meiklejohn et al., 2023 [93]	Precision ID Ancestry and Identity Panels	Illumina MiniSeq System	SNPs from dust samples were typed on the MiniSeq instrument. The FastID software was used for mixture deconvolution; 72% of the alleles were recovered, and 93% of known occupants were detected in at least one sample, while 54% had non-occupant alleles.
Themudo et al., 2016 [94]	HID-Ion AmpliSeq Ancestry Panel	Ion Torrent PGM	Ancestry Panel profiles were obtained from Greenlanders. The training set was used as reference for subsequent, more successful assignment of ancestry.
Garcia et al., 2017 [95]	Precision ID Ancestry Panel	Ion Torrent PGM	Forensic evaluation of the Ancestry Panel on Ion PGM. Population data for Basques were obtained and used for ancestry inference, which showed clustering with Europeans.
Truelsen et al., 2017 [96]	Precision ID Ancestry Panel	Ion Torrent PGM	Middle East populations (Turks and Iranians) were analyzed to investigate genetic differentiation using GenoGeographer program. Differentiation was not possible.
Pereira et al., 2017 [97]	Precision ID Ancestry Panel	Ion Torrent PGM	Evaluation of the panel with casework samples (blood and buccal swabs on FTA cards) from Somalia and Denmark was reported. Some markers consistently performed poorly.
Santangelo et al., 2017 [98]	Precision ID Ancestry Panel	Ion Torrent PGM	Ancestry and admixture investigation of three Ecuadorians ethnic groups (Kichwa, Mestizo, and Afro-Ecuadorian) was reported.
Hollard et al., 2017 [99]	HID-Ion AmpliSeq Ancestry Panel	Ion Torrent PGM	Ancestry kit, Yfiler Plus, mtDNA HV1 sequencing, and Irisplex SNPs were used to analyze ancestry and phenotype from a carbonized corpse that failed CE STR testing. A combined approach appeared preferable.
Wang et al., 2018 [100]	Precision ID Ancestry Panel	Ion Torrent PGM	Forensic evaluation, population data, and ancestry inference for Chinese Tibeto-Burman were reported. The panel performed well and was suitable for both ancestry and identification.
Nakanishi et al., 2018 [101]	Precision ID Ancestry Panel	Ion Torrent PGM	Japanese populations from mainland and Okinawa were analyzed to evaluate the possibility to distinguish them via ancestry investigation. Differentiation was not possible.
Jin et al., 2018 [102]	Precision ID Ancestry Panel	Ion S5 System	Development of guidelines for the implementation of a biogeographic ancestry inference service based on Admixture Prediction produced in the Ion Torrent Suite for Precision ID Ancestry Panel. The panel appeared effective for ancestry prediction.
He et al., 2018 [103]	Precision ID Ancestry Panel	Ion Torrent PGM	Chinese populations (Uyghur and Hui) were analyzed to investigate population data and ancestry. Individuals could be differentiated both by ancestry and individually. A limitation was observed in distinguishing homogeneous populations.
Al-Asfi et al., 2018 [104]	Precision ID Ancestry Panel	Ion Torrent PGM	Evaluation of the kit on Ion PGM. Ancestry prediction was accurate at 125 pg and 30 pg using 21 and 25 PCR cycles, respectively. Partial profiles (85%) were obtained from 15 pg of DNA, while <6 pg produced less than 50% concordance; 1 ng was suggested as minimum input for high-confidence ancestry assignment.
Lee et al., 2018 [105]	Precision ID Ancestry Panel	Ion S5 XL System	Seven Asian populations (Southern Chinese, Beijing Chinese, Japanese, Koreans, Vietnamese, Nepalese, Indians, and Pakistani) were profiled, and ancestry was investigated. All Northeast (China, Japan, and Korea) and Southeast Asians (Vietnamese) were predicted as East Asians, while Southwest Asians (Nepal, India, and Pakistan) were predominantly assigned to South Asia.
Young et al., 2019 [106]	Precision ID Ancestry Panel	Ion GeneStudio S5 System	Ancestry Panel, 24 SNP HIrisplex System, and QIAGEN 140-SNP forensic identification multiplex kits were used on different touched items. Accurate calls were obtained from 70% of samples for all three panels combined.
Daniels-Higginbotham et al., 2019 [107]	Precision ID Ancestry Panel	Illumina MiSeq FGx System	19th century skeletal remains were sequenced using MiSeq FGx to identify ancestry. AmpFℓSTR Yfiler PCR Amplification Kit and Y-typing of four important SNP variants were also used to upload results on the FamilyTreeDNA website.
Shan et al., 2019 [108]	Precision ID Ancestry Panel	N/A	Ancestry investigation from Punjabi Pakistani samples was reported. The GenoGeographer software was used. The population appeared admixed and non-distinguishable from South Central Asia and Middle East populations.
Al-Dosari et al., 2019 [109]	Precision ID Ancestry Panel	Ion Torrent PGM	Comparison between manual and automated library preparation to type casework samples. No significant differences were found, but the automated method was faster and avoided pipetting errors. A full profile was obtained from 0.12 ng of DNA.
Mogensen et al., 2020 [110]	Precision ID Ancestry Panel	N/A	The GenoGeographer software was assessed for ancestry assignment using publicly available data from multiple populations. Ancestry was not assigned to 22.4% of the individuals. Of the assigned individuals, 8.2% had discordant assignment, and 8.2% had ambiguous assignment. Prediction would be improved with more SNPs and more data in databases.
Cooley et al., 2021 [111]	Precision ID Ancestry Panel	Ion S5 System and Ion Torrent PGM	Comparison of Ion OneTouch 2/Ion PGM and Ion Chef/Ion S5 systems using forensic-type samples. The Ion Chef/Ion S5 method was faster and generated higher coverage and SNP quality, even if both systems predicted concordant ancestries.
Truelsen et al., 2021 [112]	Precision ID Ancestry Panel	Ion S5 System	Samples from 14 European, Middle Eastern, African, and Asian countries were sequenced with the Ancestry and EUROFORGEN panels to perform ancestry prediction with GenoGeographer. The Ancestry Panel was sufficient to distinguish North Africans from European and Middle Eastern, with improved separation when combining the two panels. Separation of Middle Eastern from Europeans and South-Central Asians was difficult even with both kits combined.
He et al., 2021 [113]	Precision ID Ancestry Panel	Ion S5 XL System	Population genetics, admixture, ancestry, and forensic parameters were evaluated in Southern Chinese Sinitic/Tai-Kadai individuals.
Shan et al., 2021 [114]	Precision ID Ancestry Panel	Ion S5 System	Pakistani individuals (Baloch, Pashtun, and Punjabi subpopulations) samples were processed with the Ancestry Panel and HuPi AmpliSeq Custom panel to evaluate both ancestry and skin pigmentation. Significantly different genetic distance was observed between the subgroups. Skin differentiation was also significantly different for one group compared to the other two.
Young et al., 2021 [115]	Precision ID Ancestry Panel	Ion GeneStudio S5 Plus System	The Ancestry Panel and HIrisplex System (Ion AmpliSeq HID Phenotyping Community Panel) were used for ancestry evaluation and skin pigmentation prediction applied to touch DNA samples with direct PCR. 90% of samples generated correct ancestry assignment at the major population level. Full SNP profiles were obtained from 23% of touch samples and partial profiles (>85%) from 32%. Moreover, 42% of samples obtained high confidence ancestry assignments. Correct ancestry was assigned when >70% SNPs were detected.
Mogensen et al., 2022 [116]	Precision ID Ancestry Panel	N/A	Data for Slovenia, Greece, Albania, and Eritrea were obtained and added to GenoGeographer as a reference population. The Admixture Module was tested on 3548 profiles; 95.5% were assigned to one or more groups (95.4% concordant), while 4.5% were not assigned. An additional 1486 profiles expected to belong to populations other than the reference were also tested; 70% of North and South America samples were rejected, while only 20% of Central, North, and Northeast Asia samples were rejected. The rejection rate was decreased by using the Admixture Module.
Cui et al., 2023 [117]	Precision ID Ancestry Panel	Ion S5 XL System	Gannan Tibetan population samples were analyzed, and forensic parameters and ancestry were evaluated. The kit appeared useful for ancestry prediction for continental populations but less accurate for subpopulations. Individual identification appeared suitable.
Felkl et al., 2023 [118]	HID-Ion AmpliSeq Ancestry Panel	Ion Torrent PGM	Forensic evaluation and ancestry prediction of South Brazilian heterogeneous population samples were performed. Use for individual identification and kinship appeared suitable. Ancestry analysis showed admixture and more homogeneous groups among the subtypes.
Koksal et al., 2023 [119]	Precision ID Ancestry Panel	Ion S5 System	Ancestry and admixture investigation of Brazilians using GenoGeographer was reported. Performance was low on the heterogeneous Brazilian population; 55% of assignments failed. Likelihood Ratio (LR) < 1000 was observed as a possible indicator of admixture. Higher Asian and African genetic contributions were observed in failed samples.
Huang et al., 2018 [120]	N/A	N/A	A panel of 117 universal SNPs with Minor Allele Frequency (MAF) > 0.39 in 37 populations was defined for identity testing. Combined Match Probability (CMP) was lower for this set rather than the HID-Ion AmpliSeq Identity Panel for the Chinese Han population.
Ragazzo et al., 2021 [121]	N/A	N/A	Evaluation of a custom OpenArray panel for phenotype prediction and identification including 60 SNPs from HIrisplex-s, Precision ID Identity SNP Panel, and ForenSeq DNA Signature Prep Kit.
Resutik et al., 2023 [122]	N/A	N/A	Ancestry prediction performance of MAPlex, Precision ID Ancestry Panel, and VISAGE Basic Tool panels was compared using publicly available datasets. Similar results were produced from all panels when using six broad continental regions on STRUCTURE. The most consistent performance in all regions was given by the VISAGE panel.

**Table 2:** List of populations screened in the reviewed publications.

Continent	Country	Population	Publication/s
Asia	Afghanistan	Afghani	Truelsen et al., 2021 [112]
	China	Beijing Chinese	Lee et al., 2018 [105]
		Chinese Han	Wang et al., 2017 [35], Guo et al., 2016 [57], Sun et al., 2019 [71], Wang et al., 2020 [45]
		Chinese Hui	He et al., 2018 [103], Liu et al., 2018 [69]
		Chinese Gannan Tibetan	Cui et al., 2023 [117]
		Chinese Tibetans	Wang et al., 2020 [45], Liu et al., 2018 [69]
		Chinese Tibeto-Burman	Wang et al., 2018 [100]
		Chinese Uyghur	He et al., 2018 [103], Tao et al., 2019 [37], Liu et al., 2018 [69]
		Southern Chinese	Li et al., 2018 [70], Lee et al., 2018 [105]
		Southern Chinese Sinitic/Tai-Kadai	He et al., 2021 [113]
	India	Central Indians	Dash et al., 2021 [47], Dash et al., 2022 [83]
		Indians	Lee et al., 2018 [105]
	Iran	Iranians	Truelsen et al., 2017 [96], Truelsen et al., 2021 [112]
	Iraq	Iraqi	Truelsen et al., 2021 [112]
	Japan	Japanese	Kitayama et al., 2022 [48], Ohuchi et al., 2022 [49], Nakanishi et al., 2018 [101], Ochiai et al., 2016 [58], Lee et al., 2018 [105]
	Korea	Koreans	Yang, Lee and Lee, 2023 [85], Lee et al., 2018 [105]
	Malaysia	Malay	Ochiai et al., 2016 [58]
	Myanmar	Burmese	Joo et al., 2023 [86]
	Nepal	Nepalese	Lee et al., 2018 [105]
	Pakistan	Pakistani	Lee et al., 2018 [105], Truelsen et al., 2021 [112]
		Pakistani Baloch	Shan et al., 2021 [114]
		Pakistani Pashtun	Shan et al., 2021 [114]
		Pakistani Punjabi	Shan et al., 2019 [108], Shan et al., 2021 [114]
	Syria	Syrians	Truelsen et al., 2021 [112]
	Turkey	Turks	Truelsen et al., 2017 [96], Truelsen et al., 2021 [112]
	Vietnam	Vietnamese	Lee et al., 2018 [105]
Europe	Albania	Albanians	Truelsen et al., 2021 [112], Mogensen et al., 2022 [116]
	Denmark	Danes	Truelsen et al., 2021 [112], Buchard et al., 2016 [59], Pereira et al., 2017 [97]
	Greece	Greeks	Truelsen et al., 2021 [112], Mogensen et al., 2022 [116]
	Portugal	Portuguese	Truelsen et al., 2021 [112]
	Slovenia	Slovenians	Truelsen et al., 2021 [112], Mogensen et al., 2022 [116]
	Spain	Basques	Garcia, Soto and Yurrebaso, 2017 [64], Garcia et al., 2017 [95]
		Spanish	Barrio et al., 2019 [43]
Africa	Eritrea	Eritreans	Truelsen et al., 2021 [112], Mogensen et al., 2022 [116]
	Morocco	Moroccans	Truelsen et al., 2021 [112]
	Somalia	Somali	Truelsen et al., 2021 [112], Pereira et al., 2017 [97], van der Heijden et al., 2017 [63]
South America	Brazil	Brazilians	Bottino, Silva and Moura-Neto, 2019 [74], Avila et al., 2019b [75], Koksal et al., 2023 [119]
		South Brazilians	Felkl et al., 2023 [118]
	Ecuador	Ecuadorians	Santangelo et al., 2017 [98]
North America	Greenland	Greenlanders	Themudo et al., 2016 [94]

## Data Availability

No new data were created during the preparation of this manuscript.
